# Simultaneous Mutations in Multi-Viral Proteins Are Required for *Soybean mosaic virus* to Gain Virulence on Soybean Genotypes Carrying Different *R* Genes

**DOI:** 10.1371/journal.pone.0028342

**Published:** 2011-11-30

**Authors:** R. V. Chowda-Reddy, Haiyue Sun, John H. Hill, Vaino Poysa, Aiming Wang

**Affiliations:** 1 Southern Crop Protection and Food Research Centre, Agriculture and Agri-Food Canada, London, Ontario, Canada; 2 Department of Biology, University of Western Ontario, London, Ontario, Canada; 3 Department of Plant Pathology, Iowa State University, Ames, Iowa, United States of America; 4 Greenhouse and Processing Crops Research Centre, Agriculture and Agri-Food Canada, Harrow, Ontario, Canada; Nanjing Agricultural University, China

## Abstract

**Background:**

Genetic resistance is the most effective and sustainable approach to the control of plant pathogens that are a major constraint to agriculture worldwide. In soybean, three dominant *R* genes, i.e., *Rsv1*, *Rsv3* and *Rsv4*, have been identified and deployed against *Soybean mosaic virus* (SMV) with strain-specificities. Molecular identification of virulent determinants of SMV on these resistance genes will provide essential information for the proper utilization of these resistance genes to protect soybean against SMV, and advance knowledge of virus-host interactions in general.

**Methodology/Principal Findings:**

To study the gain and loss of SMV virulence on all the three resistance loci, SMV strains G7 and two G2 isolates L and LRB were used as parental viruses. SMV chimeras and mutants were created by partial genome swapping and point mutagenesis and then assessed for virulence on soybean cultivars PI96983 (*Rsv1*), L-29 (*Rsv3*), V94-5152 (*Rsv4*) and Williams 82 (*rsv*). It was found that P3 played an essential role in virulence determination on all three resistance loci and CI was required for virulence on *Rsv1*- and *Rsv3*-genotype soybeans. In addition, essential mutations in HC-Pro were also required for the gain of virulence on *Rsv1*-genotype soybean. To our best knowledge, this is the first report that CI and P3 are involved in virulence on *Rsv1-* and *Rsv3*-mediated resistance, respectively.

**Conclusions/Significance:**

Multiple viral proteins, i.e., HC-Pro, P3 and CI, are involved in virulence on the three resistance loci and simultaneous mutations at essential positions of different viral proteins are required for an avirulent SMV strain to gain virulence on all three resistance loci. The likelihood of such mutations occurring naturally and concurrently on multiple viral proteins is low. Thus, incorporation of all three resistance genes in a soybean cultivar through gene pyramiding may provide durable resistance to SMV.

## Introduction

Plant pathogens, causal agents of numerous devastating crop diseases worldwide, are a major constraint to agriculture and threaten global food security [1]. The use of genetic resistance is considered the most effective and sustainable approach to the control of plant pathogens as it is environmentally-friendly and target-specific, and provides reliable protection without additional labor or material costs during the growing season [2], [3]. The major genetic resistance that has been extensively used in agriculture is mediated by *R* gene. Such resistance, particularly mediated by natural dominant NBS-LRR *R* genes is triggered by either direct or indirect interactions between the *R* gene encoded protein of the host and the avirulence factor produced by the corresponding avirulence (*Avr*) gene of the invading pathogen [4]–[6]. Two defense responses, i.e., extreme resistance (ER) and hypersensitive response (HR), are often associated with *R* gene-mediated resistance [7]. In the case of plant viruses, the former is characterized by the arrest of the invading virus at the inoculation site without any visible symptoms or virus accumulation, whereas the latter restricts the virus to the primary infection site by rapid death of infected and neighboring cells [3], [7]. During the coevolutionary arms race of viral pathogens and their host plants, genetic diversity generated by spontaneous mutations (resulting from error-prone replication) and RNA recombination, and the selection force acting on this variability lead to the occurrence of resistance-breaking isolates [3], [8]–[10]. Molecular identification and characterization of virulent determinants from these isolates and their interactions with major resistance genes will advance knowledge of resistance durability, which is essential for developing and utilizing genetic resistance for crop protection [2], [7], [11].

**Figure 1:**
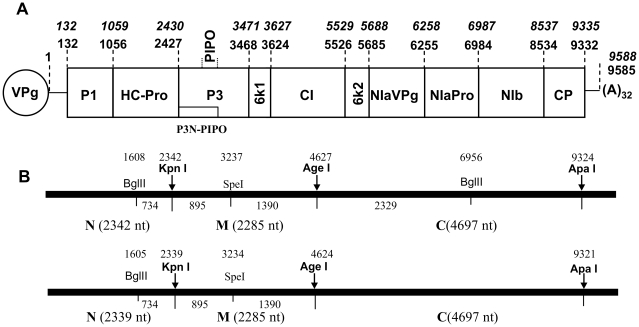
Schematic representation of three full-length infectious clones derived from SMV strains G7 and two G2 isolates, L and LRB. (A). Genomic organization of wild parental viral genomes with their respective viral proteins. Nucleotide position numbers for predicted mature proteins are indicated: italic for G7 and normal for L or LRB. (B). Restriction maps of G7 (top) and L or LRB (bottom). Arrows indicate the division of three cDNA fragments N, M and C and their nucleotide lengths are provided in parentheses. The length of the nucleotides between enzyme sites is given between those sites.

*Soybean mosaic virus* (SMV), a member of the family *Potyviridae*, is the most common viral pathogen of soybean [12]. SMV is a seed-borne, aphid-transmitted virus that causes severe yield loss and reduction in seed quality worldwide [13]. Similar to other potyviruses, SMV has a positive-sense, single-stranded RNA molecule as its genome, which is approximately 9,600 nucleotides in length encoding a large polyprotein of ∼350 kDa and a short polyprotein as a result of translational frameshift in the P3 coding region (Fig. 1A). These two polyproteins are processed by three viral proteases (P1 and HC-Pro responsible for autocleavage at their N-terminus and NIA-Pro for all other cleavages) to release 11 mature proteins, from the N-terminus: P1, HC-Pro, P3, P3N-PIPO (resulting from translational slippage or frameshift in P3), 6K1, CI, 6K2 (or 6K), NIa-VPg, NIa-Pro, NIb, and CP [14], [15]. To date, numerous SMV isolates have been reported. In North America, SMV isolates are classified into seven distinct strains, G1 through G7, based on their differential responses on susceptible and resistant soybean cultivars [16]. Screening for resistant soybean germplasm to SMV has identified three independent resistance loci, *Rsv1*, *Rsv3* and *Rsv4* [17]–[20]. These three loci are all dominant *R* genes [5], [18], [21], [22]. *Rsv1*, found in PI96983 confers extreme resistance to most SMV strains but not to G7, whereas *Rsv3*-genotype soybean is resistant to higher numbered strain groups including G5 through G7 but susceptible to lower numbered strain groups (G1 through G4) [23]. *Rsv4* is the only gene that confers resistance to all the seven strains [12].

The strain-specific *Rsv1-* and *Rsv3*-conferred resistance to SMV is associated with ER and HR, respectively [12], [24]. However, the resistance mechanism of *Rsv4* seems different as it is not associated with either ER or HR [12]. As one of the most complex plant-pathogen interactions, the soybean-SMV pathosystem is an excellent model to study R-Avr recognitions. Disturbance of R-SMV interactions can result in escape and spread of the virus to distant tissues. For instance, continued challenge of *Rsv1*-genotype soybean by SMV isolate N, a G2 isolate, induces a systemic HR (SHR), rather than HR [25]. SHR might be a consequence of delayed occurrence of HR-associated events [26]. Infection of *Rsv1*-genotype soybean by SMV strain G7, however, triggers a lethal SHR (LSHR), likely due to rapid progression of SHR [27], [28].

In the past several years, many naturally and experimentally evolved SMV resistance-breaking isolates (all the three resistance loci) were documented [3], [8], [11], [27], [29], [30]. As there are only three naturally-occurring resistant sources deployed for soybean breeding programs worldwide, concerns have been raised about the durability of these resistance genes [13]. The great majority of recent studies have focused on the genetic basis of SMV virulence on resistance mediated by each individual resistance gene. Through comparative genomic analyses, virulence proteins responsible for breaking down resistance have been mapped to HC-Pro, P3 and CI depending on the type of resistance [3], [11], [28], [31]–[33].

In this report, SMV strains G7 and G2 (isolates L and LRB) were used as parental viruses to study the gain and loss of SMV virulence on all three resistance loci. SMV chimeras and mutants were created by partial genome swapping and point mutagenesis and then assessed for virulence on soybean cultivars containing different resistance genes. We found that multiple viral proteins participated in virulence on each of the three resistance loci. Based on this study, we suggest that incorporation of all three resistance genes into a soybean cultivar through gene pyramiding may provide durable resistance to SMV.

## Results

### Virulence on *Rsv1*-, *Rsv3*- and *Rsv4*-genotype soybeans is determined by multi-viral proteins

**Figure 2:**
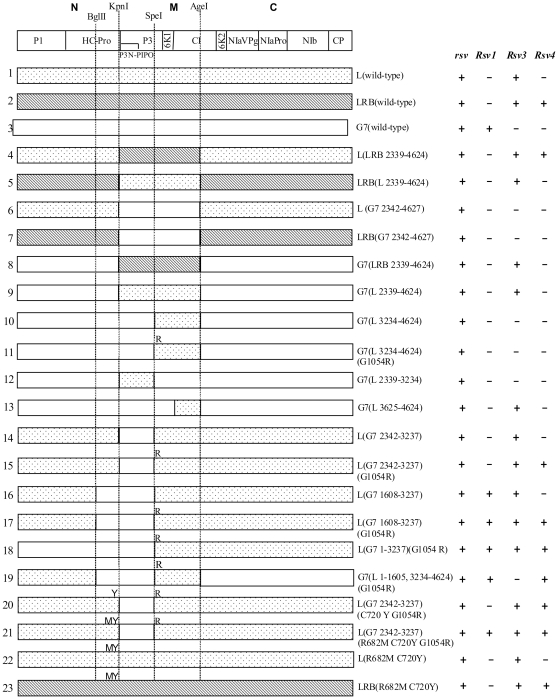
Pathogenicity assays of parental SMV infectious clones L, LRB and G7, chimeric clones and mutants with *rsv*-, *Rsv1*-, *Rsv3*- and *Rsv4* -genotype soybeans. The infectivity of the clones is shown to the right. *rsv*, Williams 82 (carrying no resistance gene); *Rsv1*, PI96983; *Rsv3*, L29; *Rsv4*, V94-5152; +, positive in ELISA and RT-PCR assays; –, negative in ELISA and RT-PCR assays.

**Figure 3:**
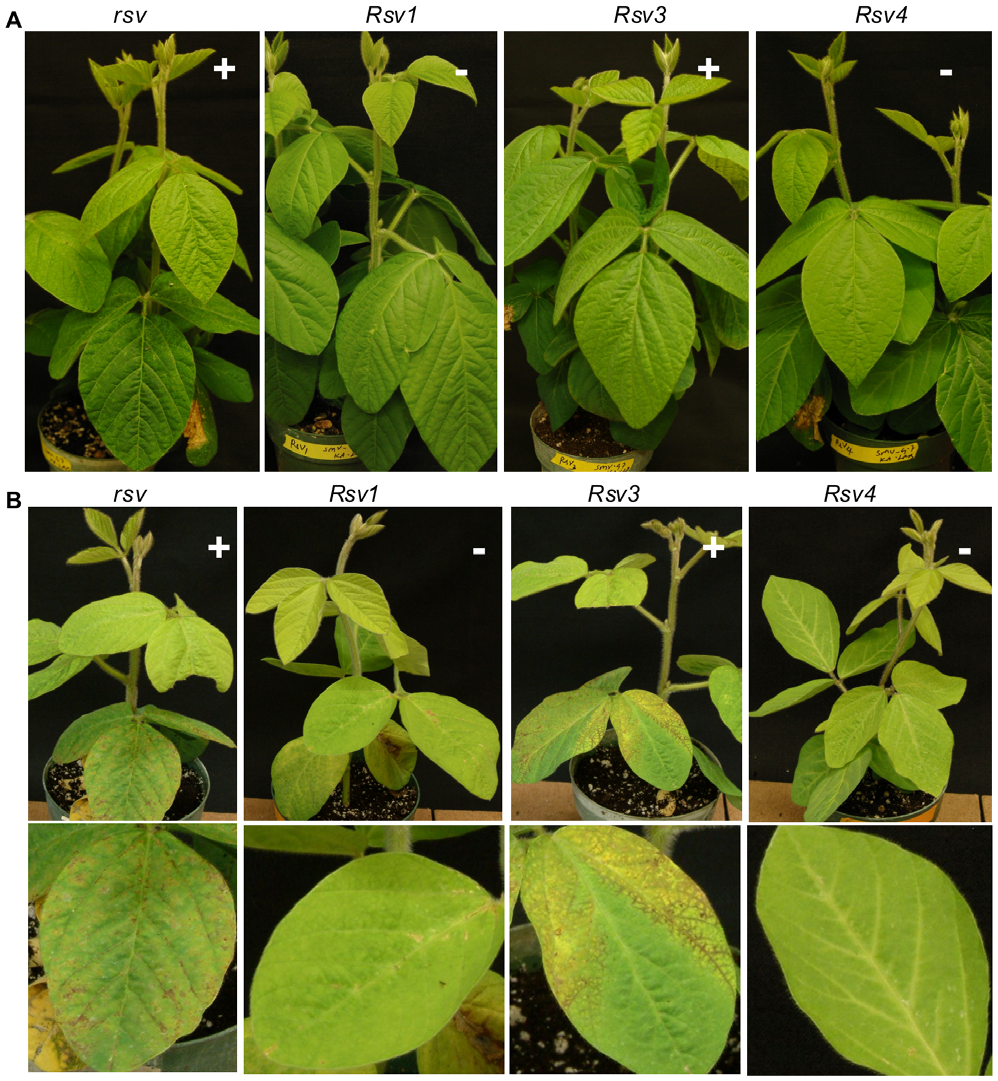
Infectivity and symptoms of soybean inoculated with chimeric SMV clones G7(L 2339-4624) and G7(L 3625-4624). (A). Inoculated with G7(L 2339-4624). Photos were taken 28 days post inoculation. (B). Inoculated with G7(L 3625-4624). Trifoliate leaves are shown underneath. Phtos were taken 21 days post inoculation. Symptoms are evident on *rsv*- and *Rsv3*-genotype soybeans. *rsv*, Williams 82 (carrying no resistance gene); *Rsv1*, PI96983; *Rsv3*, L29; *Rsv4*, V94-5152; +, positive (ELISA and RT-PCR); –, negative (ELISA and RT-PCR).

To study genetic determinants of SMV virulence on all three identified resistance loci in soybean, we used SMV strain G7 and two isolates of the G2 strain, L and LRB (Fig. 1). G7 is avirulent on *Rsv3*- and *Rsv4*-genotype soybeans and virulent on *Rsv1*-genotype soybean [12], [22]. In contrast, L infects *Rsv3*- but not *Rsv1*- and *Rsv4*-genotype soybeans, and LRB, a naturally evolved isolate of L differentiated from L by overcoming *Rsv4*-mediated resistance [8]. The full-length cDNA infectious clones derived from these three SMVs shared pathogenicity similar to their respective parental viruses (Fig. 2). Since P3 is a virulence determinant for *Rsv1*- and *Rsv4*-resistance [11], [28] and CI is critical for *Rsv3*-resistance [3], [32], we first constructed hybrid SMVs by swapping the genomic fragment M (encoding the C-terminal 30 amino acids of HC-Pro, P3, P3-PIPO, 6K1 and the N-terminal two thirds of CI) (Fig. 2). The resulting hybrid SMVs were subjected to pathogenicity assays. Reciprocal exchange of the M fragment of L and LRB did not change their avirulence on *Rsv1*-genotype and virulence on *Rsv3*-genotype soybean but resulted in the gain or loss of virulence on *Rsv4*-genotype soybean, consistent with our published data that P3 of LRB is responsible for breaking down *Rsv4*-mediated resistance [11]. When the M fragment of L or LRB was replaced with that of G7, the resulting chimeras were avirulent on *Rsv1*-, *Rsv3*- and *Rsv4*-genotype soybeans (Fig. 2). In agreement with previous observations, both the N-terminal P3 (overlapping with P3N-PIPO) and HC-Pro of G7 are required for a G2 isolate to gain virulence on *Rsv1*-genotype soybean [28], [31] and the CI of G2 is required for G7 to gain virulence on *Rsv3*-genotype soybean [32]. Chimeric SMVs derived from G7 whose M fragment was substituted with the homologous region of L or LRB lost virulence on *Rsv1*-genotype soybean, gained virulence on *Rsv3*-genotype soybean and were avirulent on *Rsv4*-genotype soybean (Figs. 2 and 3A). The loss of the G7 P3 may account for losing virulence on *Rsv1*-genotype soybean [28], [32]. The gain of virulence on *Rsv3*-genotype soybean was not expected as these hybrid SMVs did not contain *Rsv3* pathogenetic determinants identified previously, i.e., the C-terminal CI of G7H [3] or both the N- and C-terminal CI of G2 [32]. Since the LRB P3 is responsible for the gain of virulence on *Rsv4* soybean [11], it was surprising that G7(LRB 2339-4624), a G7 derivative containing the entire LRB P3, was unable to infect *Rsv4*-genotype soybean (Figs. 2 and 3A). Thus, it is reasonable to suggest that, in addition to P3, other viral protein(s) or domain(s) of G2 are also required for virulence on *Rsv4*-conferred resistance. Taken together these results suggest multi-viral proteins constitute virulence determinants for each of the three resistance loci in soybean.

### P3 is involved in virulence on all three resistance loci and CI is essential for breaking down *Rsv1*- and *Rsv3*-resistances

**Figure 4:**
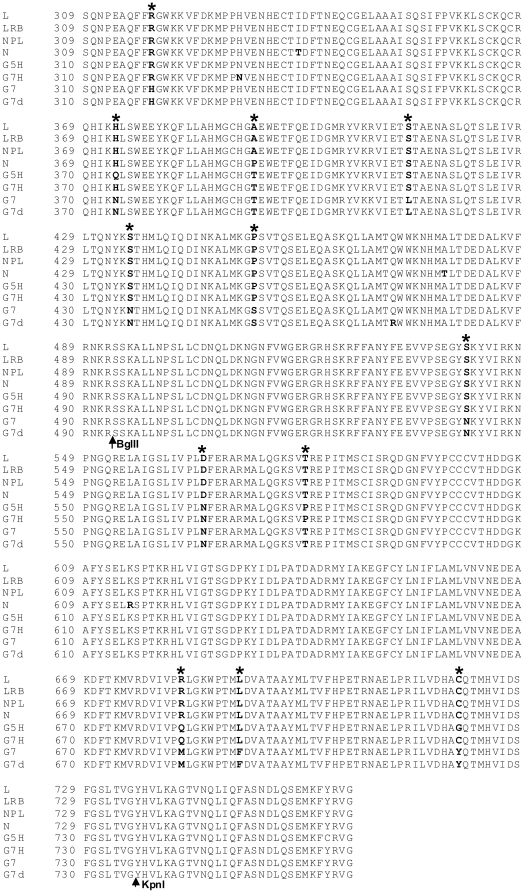
Amino acid sequence alignment of the SMV HC-Pro protein. Arrow indicates restriction sites BglII and KpnI which were used for plasmid construction. Numbers are the amino acid positions of the deduced polyprotein encoded by the long open reading frame. As shown, the last 30 amino acid sequence (after KpnI) is identical between G7 and G2 strains. The position numbers of two point mutations in this study are indicated.

**Figure 5:**
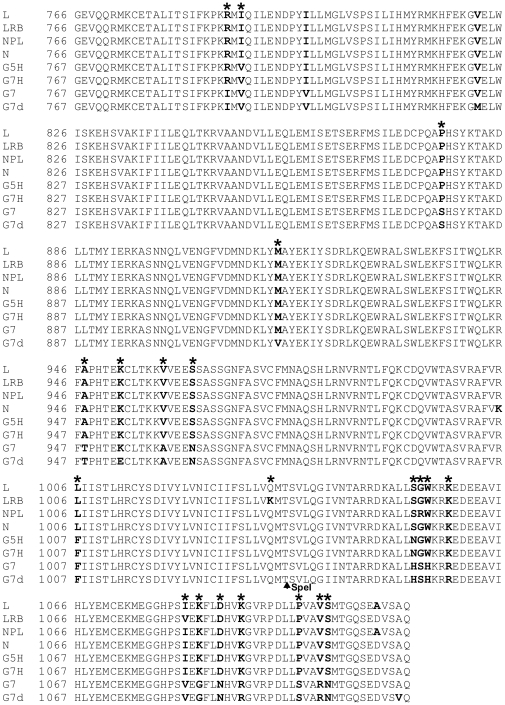
Amino acid sequence alignment of the SMV P3 protein. Restriction site SpeI used for clone construction is shown. * indicates two essential amino acids K and R responsible for breaking down *Rsv4*-mediated resistance.

The amino acid sequence of HC-Pro, P3, P3N-PIPO, 6K1 and CI was compared among SMV isolates. The very C-terminal HC-Pro amino-acid sequence (downstream of the KpnI site) consisting of 30 amino acids, is identical among all the SMV isolates analyzed including G2 and G7 isolates (Fig. 4). The 6K1 sequence is also highly conserved among SMV isolates with only one substitution (A to V) concerning two similar amino acids for two isolates (Fig. S1). Therefore, these two regions are unlikely to be virulence determinants on the three resistance loci. To further determine the virulence role of P3 (consisting of the embedded P3N-PIPO) (Fig. 5; Fig. S2) and the N-terminal CI (Figs. S3 and S4), two more hybrid SMVs, G7(L 3234-4624) and G7(L 2339-3234) were created (Fig. 2). Both of them lost virulence on *Rsv3*-genotype soybean (Fig. 2), indicating neither the N-terminal P3 (including P3N-PIPO) of G2 nor the C-terminal P3/N-terminal CI of G2 was sufficient for virulence on *Rsv3*-genotype soybean. Thus, both P3 and CI were involved in virulence on *Rsv3*-genotype soybean. Neither of these two viruses restored virulence on *Rsv1*-genotype soybean (Fig. 2), indicating the N-terminal P3 of G7 as well as the C-terminal P3/N-terminal CI of G7 was essential for G7 to maintain virulence on *Rsv1*-genotype soybean. Previously, a single mutation (G1054R), downstream of the KpnI site in the C-terminal P3 of isolate NPL, an L derivative (Fig. 5) was sufficient to make isolate L virulent on *Rsv4*-genotype soybean [11]. This mutation was introduced into the hybrid virus G7(L 3234-4624). The resulting virus G7(L 3234-4624)(G1054R) was unable to infect *Rsv4*-genotype soybean or other resistant soybeans (Fig. 2). This result again supports that the G2 P3 must function with other viral determinants including CI (see below) for virulence on *Rsv4*-resistance. To further clarify the virulence role of the N-terminal CI, a chimeric infectious clone G7(L 3625-4624) was constructed. In comparison with wild-type G7, this recombinant virus lost virulence on *Rsv1*-genotype soybean but gained infectivity on *Rsv3*-genotype soybean (Figs. 2 and 3B), indicating an essential role of CI for breaking down both *Rsv1*- and *Rsv3*-resistances. The fact that this chimeric virus infected *Rsv3*-genotype soybean, in comparison with recombinant clones G7(L 3234-4624) and G7(L 2339-4624), confirmed the involvement of P3 in breaking down Rsv3-resistance (Figs. 2 and 3). Taken together these data suggest P3 is involved in virulence on all three resistance loci and CI is essential at least for virulence on *Rsv-1* and *Rsv3*-genotype soybeans.

### The N-terminal P3 (including P3N-PIPO) of G2 is not essential for G2 to maintain virulence on *Rsv3*- and *Rsv4*-genotype soybeans and that of G7 is insufficient for G2 to gain virulence on *Rsv1*-genotype soybean

**Figure 6:**
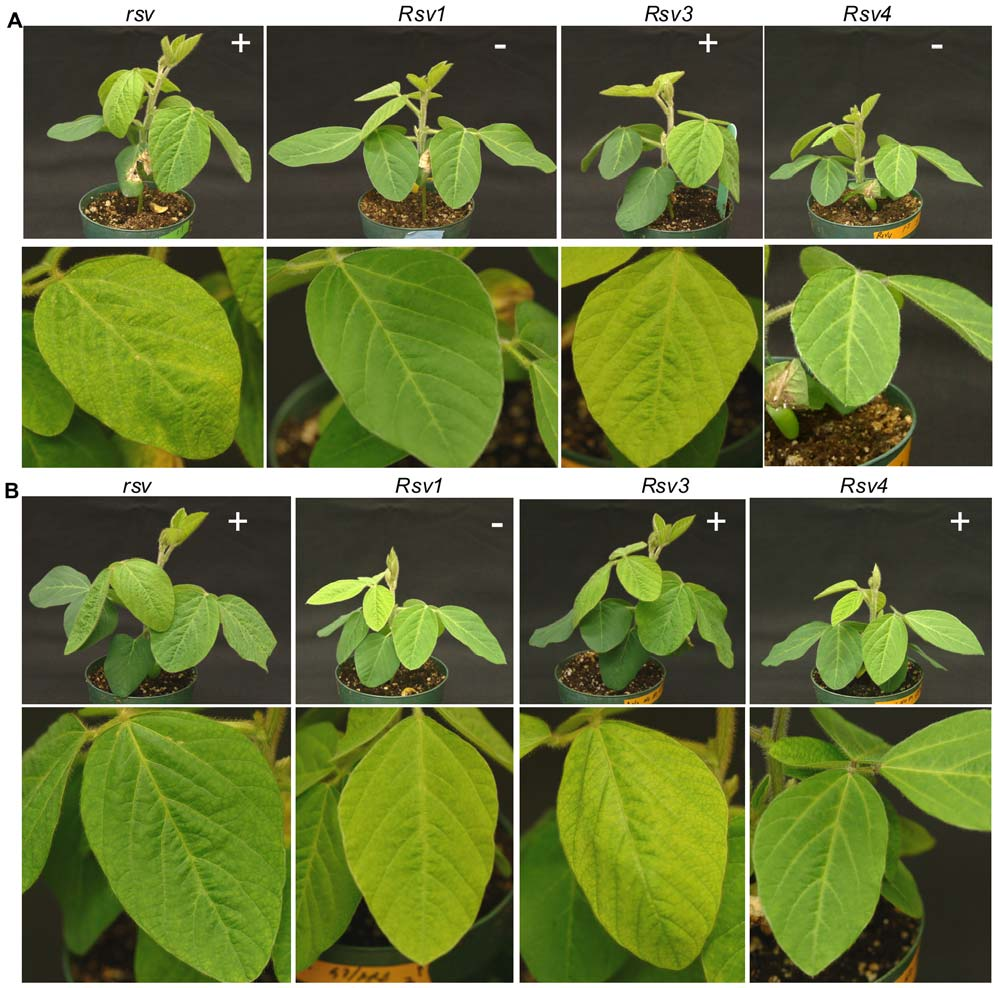
Infectivity and symptoms of soybean inoculated with chimeric SMV clones L(G7 2342-3237) and L(G7 2342-3237)(G1054R). (A). inoculated with L(G7 2342-3237). (B). Inoculated with L(G7 2342-3237)(G1054R). Trifoliate leaves are shown underneath. Photos were taken 14 days post inoculation. *rsv*, Williams 82 (carrying no resistance gene); *Rsv1*, PI96983; *Rsv3*, L29; *Rsv4*, V94-5152; +, positive (ELISA and RT-PCR); –, negative (ELISA and RT-PCR).

To test if the N-terminal P3 (including P3N-PIPO) is essential for virulence on *Rsv3*-genotype soybean, the KpnI-SpeI fragment of the L isolate and its mutant containing mutation G1054R that breaks down *Rsv4*-resistance [11] was replaced with that of G7 to generate hybrid SMVs L(G7 2342-3237) and L(G7 2342-3237)(G1054R) (Fig. 2). As mentioned above, the sequence of the very C-terminal 30 amino acids of HC-Pro downstream of KpnI was identical among G7, L, LRB and other G7 and G2 isolates (Fig. 4). Therefore, the two chimeric SMVs actually obtained about four-fifths of the P3 from the N terminus (including the entire P3N-PIPO) from G7 (Fig. 5, Fig. S2). Both hybrid SMVs retained their infectivity on *Rsv3*-genotype soybean (Figs. 2 and 6), implying the N-terminal P3 (including P3N-PIPO) of G2 was not essential for virulence on *Rsv3*-genotype soybean. All *Rsv4*-genotype soybean plants were susceptible to L(G7 2342-3237)(G1054R) (Figs. 2 and 6), indicating the N-terminal P3 of G2 was not essential for virulence on *Rsv4*-resistance. Interestingly, both chimeric SMVs acquiring the N-terminal P3 of G7 were avirulent on *Rsv1*-genotype soybean (Fig. 6) and all *Rsv4*-genotype soybeans showed resistance to L(G7 2342-3237) (Fig. 6) except for one plant. Sequencing the virus isolated from this plant revealed three amino-acid mutations, i.e., G1054R, S1804G and K2787R. Further mutation analysis showed that the G1504R mutation rather than S1804 within the 6K2 protein and K2787R in CP was responsible for breaking down *Rsv4*-resistance (data not shown). Thus, the N-terminal P3 (including P3N-PIPO) of G2 is not essential for virulence on *Rsv3*- and *Rsv4*-genotype soybeans and this region of G7 is insufficient for virulence on *Rsv1*-genotype soybean.

### The C-terminal moiety of HC-Pro and the N-terminal P3 of G7 together are sufficient for G2 to break down *Rsv1*-resistance

**Figure 7:**
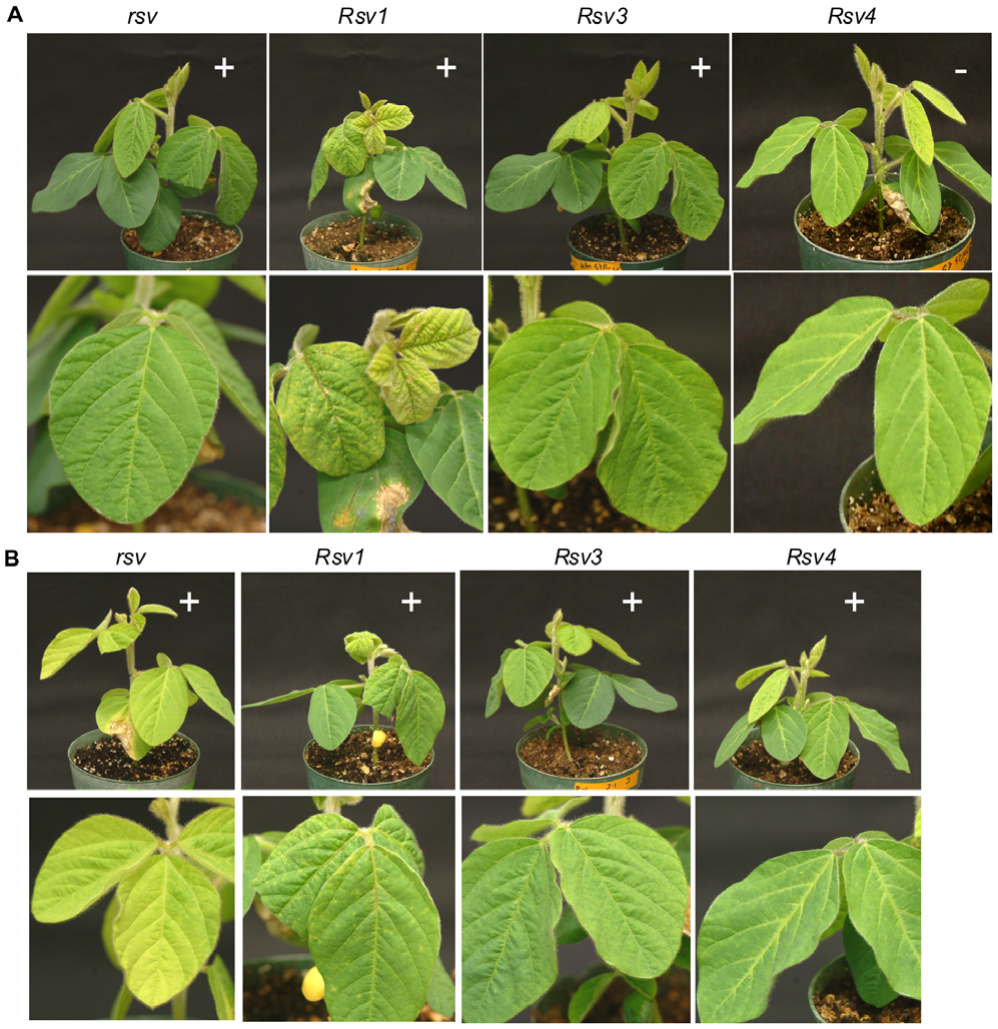
Infectivity and symptoms of soybean inoculated with chimeric SMV clones L(G7 1608-3237) and L(G7 1608-3237)(G1054R). (A). Inoculated with L(G7 1608-3237). (B). Inoculated with L(G7 1608-3237)(G1054R). Trifoliate leaves are shown underneath. Photos were taken 14 days post inoculation. *rsv*, Williams 82 (carrying no resistance gene); *Rsv1*, PI96983; *Rsv3*, L29; *Rsv4*, V94-5152; +, positive (ELISA and RT-PCR); –, negative (ELISA and RT-PCR).

**Figure 8:**
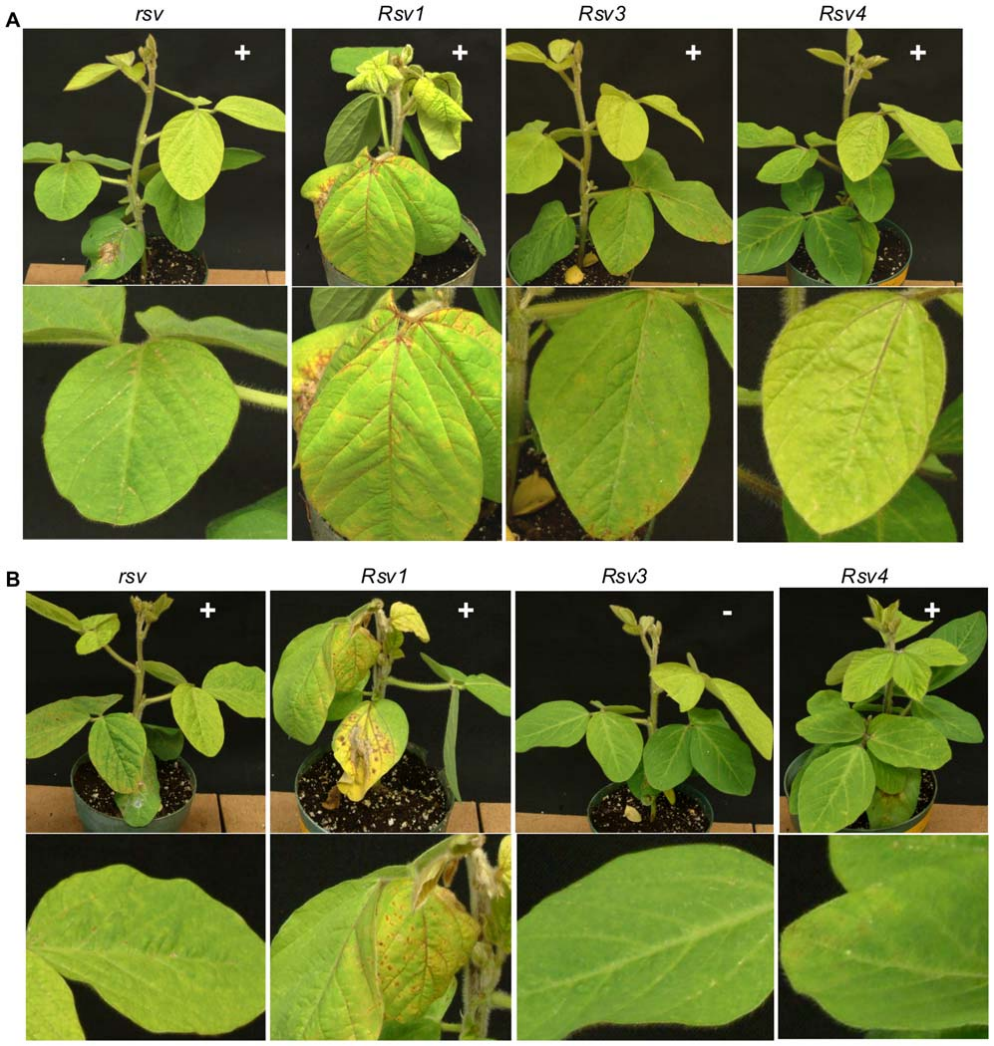
Infectivity and symptoms of soybean inoculated with chimeric SMV clones L(G7 1-3237) G1054R and G7(L1-1605)L(3234-4624) G1054R. (A). Inoculated with L(G7 1-3237) G1054R. (B). Inoculated with G7(L1-1605)L(3234-4624) G1054R. Trifoliate leaves are shown underneath. Photos were taken 3 weeks post inoculation. *rsv*, Williams 82 (carrying no resistance gene); *Rsv1*, PI96983; *Rsv3*, L29; *Rsv4*, V94-5152; +, positive (ELISA and RT-PCR); –, negative (ELISA and RT-PCR).

**Figure 9:**
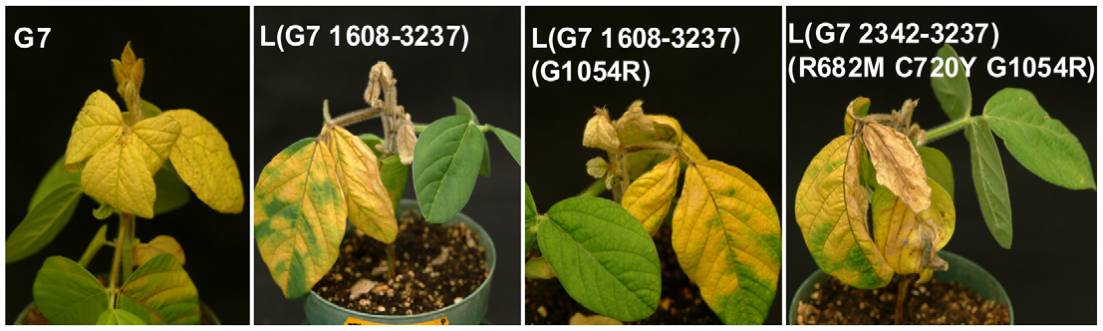
Symptoms of *Rsv1*-genotype soybean inoculated with SMV clones G7, L(G7 1608-3237), L(G7 1608-3237)(G1054R) and L(G7 2342-3237)(R682M, C720Y, G1054R). Dead leaf tissues resulting from lethal systemic hypersensitive response (LSHR) were evident on *Rsv1*-genotype soybean inoculated with all four SMVs. Photos were taken 42 days post inoculation.

The C-terminal moiety (the BglII and KpnI fragment) of HC-Pro of L(G7 2342-3237) and L(G7 2342-3237)(G1054R) was further replaced with the corresponding region of G7 to generate chimeric SMVs L(G7 1608-3237) and L(G7 1608-3237)(G1054R) (Figs. 2 and 4). Pathogenicity tests showed that L(G7 1608-3237) retained virulence on *Rsv3*-genotype soybean, remained avirulent on *Rsv4*-genotype soybean and gained virulence on *Rsv1*-genotype soybean. L(G7 1608-3237)(G1054R) retained virulence on *Rsv3*-genotype soybean and gained virulence on *Rsv1*- and *Rsv4*-genotype soybeans (Figs. 2 and 7). To test if the N-terminal virus-encoded polyprotein (upstream of BglII) and the C-terminal polyprotein (downstream of AgeI) affect virulence on *Rsv1*-, *Rsv3*- and *Rsv4*-genotype soybeans, L(G7 1608-3237)(G1054R) was used as a parental clone to produce recombinant viruses L(G7 1-3237)(G1054R) and G7(L 1-1605, 3234-4624)(G1054R). These two chimeric viruses retained infectivity on *Rsv1*- and *Rsv4*-genotype soybeans (Fig. 8), indicating either N- (upstream of BglII) or C-termini (downstream of AgeI) of the virus-encoded polyprotein between G2 and G7 does not contain avirulent determinant(s) on *Rsv1*- and *Rsv4*-resistances. However, replacement of the C-terminal polyprotein (downstream of AgeI) did affected virulence on *Rsv3*-genotype soybean (Fig. 8B), suggesting the involvement of this region in breaking down *Rsv3*-resistance. The symptoms in *Rsv1*-genotype soybean plants induced by L(G7 1608-3237), L(G7 1608-3237)(G1054R), L(G7 1-3237)(G1054R) and G7(L 1-1605, 3234-4624)(G1054R) were typical of LSHR (Figs. 7, 8 and 9), similar to those induced by G7 (Fig. 9). These results demonstrate that the C-terminal moiety of HC-Pro of G7 and the N-terminal P3 of G7 co-determine virulence on *Rsv1*-genotype soybean but the corresponding regions of G2 are not required for virulence on *Rsv3*- and *Rsv4*-genotype soybeans. As mentioned earlier, L(G7 2342-4627) or LRB(G7 2342-4624) lost virulence on *Rsv3*- and *Rsv4*-genotype soybeans and the G2 P3 must function with other viral determinant(s) to maintain virulence on *Rsv3*- and *Rsv4*-genotype soybeans. Taken together, these data suggest the G2 CI is critical for virulence on *Rsv3*-genotype soybean and likely on *Rsv4*-genotype soybean as well. Indeed, SMVs, i.e., G7(L3234-4624)(G1054R) and G7(L 2339-3234) consisting of CI hybrids (G2/G7) or the entire G7 CI abolished virulence on *Rsv3*- and *Rsv4*-genotype soybeans (Fig. 2).

**Figure 10:**
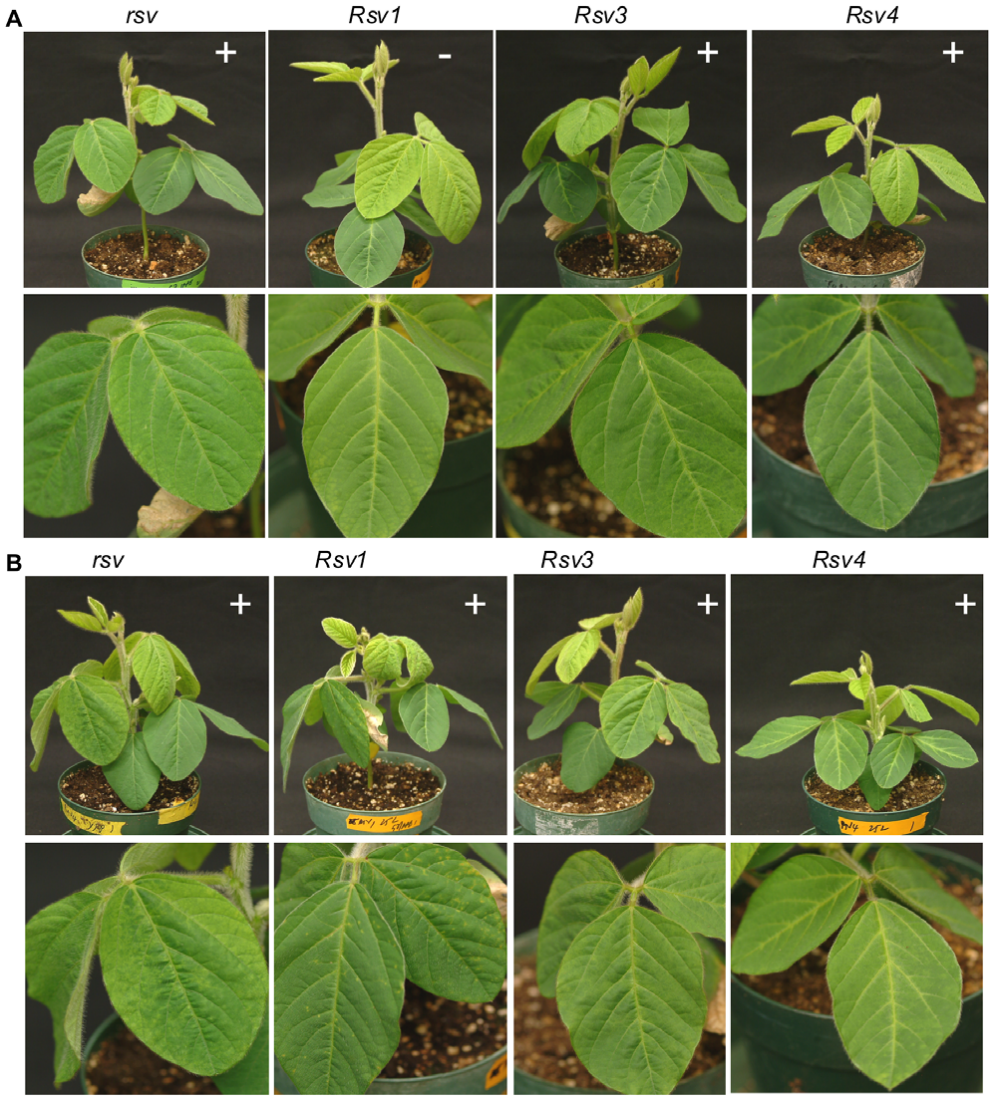
Infectivity and symptoms of soybean inoculated with chimeric SMV clones L(G7 2342-3237)(C720Y) and L(G7 2342-3237)(R682M, C720Y). (A). Inoculated with L(G7 2342-3237)(C720Y). (B). Inoculated with L(G7 2342-3237)(R682M, C720Y). Trifoliate leaves are shown underneath. Photos were taken 14 days post inoculation. *rsv*, Williams 82 (carrying no resistance gene); *Rsv1*, PI96983; *Rsv3*, L29; *Rsv4*, V94-5152; +, positive (ELISA and RT-PCR); –, negative (ELISA and RT-PCR).

**Figure 11:**
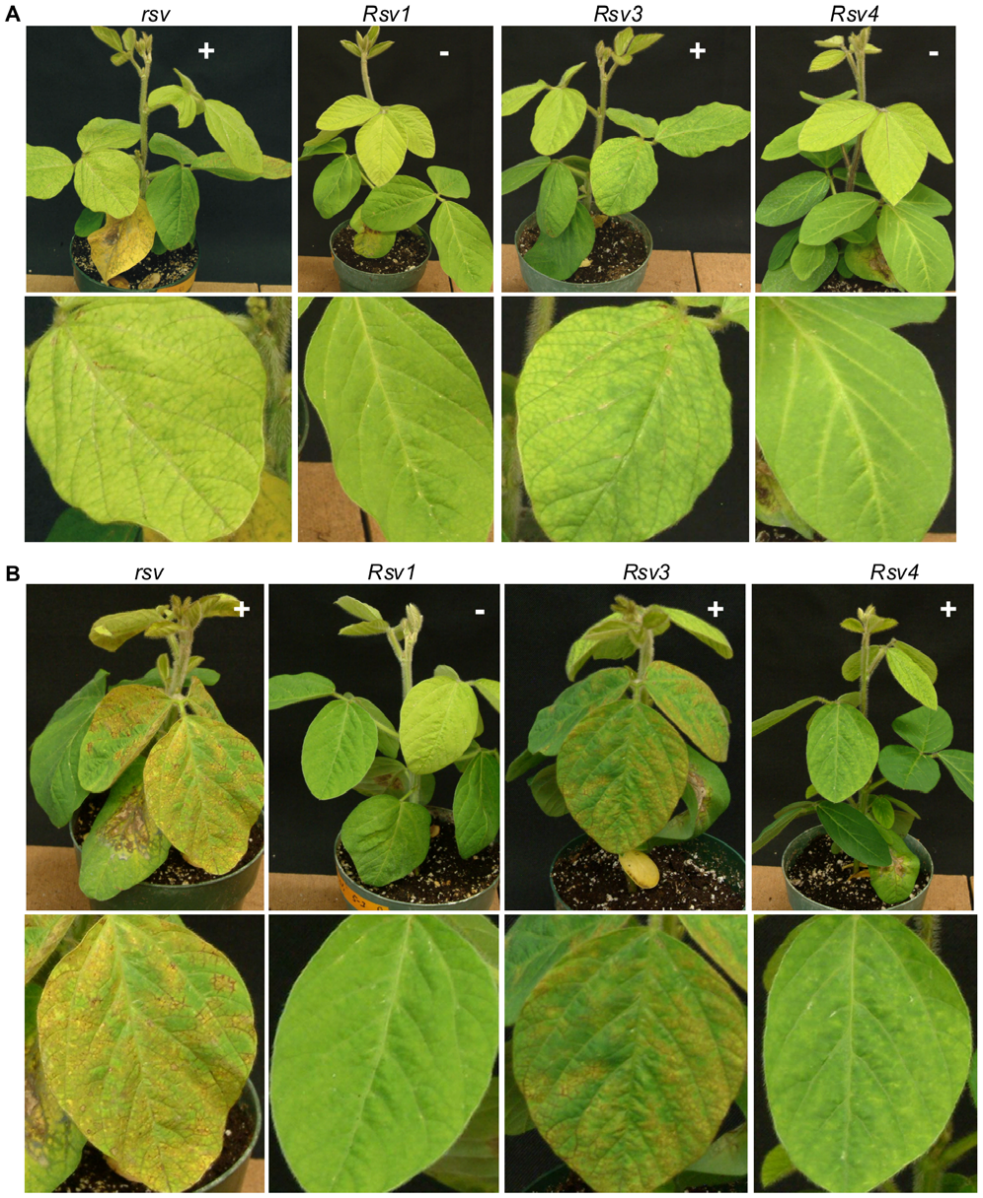
Infectivity and symptoms of soybean inoculated with chimeric SMV clones L(R682M C720Y) and LRB (R682M C720Y). (A). Inoculated with L(R682M C720Y). (B). Inoculated with LRB (R682M C720Y). Trifoliate leaves are shown underneath. Photos were taken 3 weeks post inoculation. *rsv*, Williams 82 (carrying no resistance gene); *Rsv1*, PI96983; *Rsv3*, L29; *Rsv4*, V94-5152; +, positive (ELISA and RT-PCR); –, negative (ELISA and RT-PCR).

Alignment of the C-terminal moiety of HC-Pro revealed a difference of five amino acids between L and G7. Point mutagenesis was carried out to determine if any mutations are sufficient for L(G7 2342-3237)(G1054R) to gain virulence on *Rsv1*-genotype soybean. Single (C720Y) and double mutations (R682M, C720Y) were introduced into L(G7 2342-3237)(G1054R) to produce SMVs L(G7 2342-3237)(C720Y G1054R) and L(G7 2342-3237)(R682M C720Y G1054R). L(G7 2342-3237)(C720Y G1054R) showed no symptoms or infection on *Rsv1*-genotype soybean but retained virulence on *Rsv3*- and *Rsv4*-genotype soybeans (Fig. 10), similar to L (G7 2342-3237)(G1054R). L(G7 2342-3237)(R682M C720Y G1054R), however, was not only virulent on *Rsv3*- and *Rsv4*-genotype soybeans as the case for L(G7 2342-3237)(G1054R), but also gained virulence on *Rsv1*-genotype soybean (Fig. 10). Similar to G7, L(G7 1608-3237) or L(G7 1608-3237)(G1054R), L(G7 2342-3237)(R682M C720Y G1054R) induced typical LSHR 16 or more days post inoculation (dpi) on *Rsv1*-genotype soybean (Fig. 9). To further test if these two mutations are sufficient for L(wild-type) and LRB9wild-type) to break down Rsv1-resistance, L and LRB mutants, L(R682M C720Y) and LRB (R682M C720Y), were generated. These two mutants failed to infect Rsv1-genotype soybean (Fig. 11). Taken together these data suggest that simultaneous mutations at essential residues of HC-Pro and P3 of G2 are required for the gain of virulence on *Rsv1*-genotype soybean.

## Discussion

In this study, three SMV isolates of two strains with different responses on *Rsv1*-, *Rsv3*- and *Rsv4*-genotype soybeans were employed to study virulence determinants required for virulence on soybean genotypes carrying different resistance genes. Through comparative genomic analyses, we have provided evidence that P3 is involved in virulence on these resistant cultivars. In recent studies, the potyviral P3 protein (not the embedded PIPO) has been shown as a major determinant for the loss and gain of virulence in a number of potyviral pathosystems. For instance, a mutation (A1047V) in the C-terminus (downstream of PIPO) of the *Tobacco etch virus* (TEV) P3 protein was found to be associated with the adaptation of TEV to *Arabidopsis thaliana* [34]. Multiple determinants in the N-terminal P3 (upstream of PIPO) of *Pea seed-borne mosaic virus* were identified to determine the gain and loss of virulence in *Pisum sativum* carrying the recessive resistance gene *sbm-2* [35]. The virulence determinants of *Turnip mosaic virus* (TuMV) on *Brassica napus* cultivars carrying resistance genes *TuRB03* or *TuRB04* were mapped to both the N- and C-termini of P3 (outside PIPO) [36], [37]. The N-terminal P3 (upstream of PIPO) of TuMV strain TuR1 was also shown to determine the systemic necrosis in *Arabidopsis* ecotype Ler carrying the dominant gene *TuNI* through the protein-protein interaction between TuNI and P3 [38]. In the case of SMV, the N-terminal P3 (before PIPO) of G7 was shown to be essential for its virulence on *Rsv1*-genotype soybean [39]. When the P3 or the N-terminal moiety of P3 of G7 was replaced with the corresponding region from isolates N, L and LRB (three avirulent G2 isolates), the resulting SMVs lost virulence on *Rsv1*-genotype soybean [39]. We have also reported that the C-terminal P3 (after PIPO) of isolate LRB, a naturally evolved G2 isolate, is responsible for breaking down *Rsv4*-mediated resistance [11]. A point mutation in the C-terminal P3 (after PIPO) in the isolate L (Q1033K or G1054R) [11] or recombinant SMVs L(G7 2342-3237) (G1054R) and L(G7 1608-3237)(G1054R) in this study is sufficient to alter pathogenicity on *Rsv4*-genotype soybean. In this report, we have shown that a recombinant G7 containing the entire P3 as well as the N-terminal CI of L, a G2 isolate, gained virulence on *Rsv3*-genotype soybean (Figs. 2 and 3A). When the N-terminal P3 was reversely swapped back to the G7 type, the resulting recombinant SMV G7(L 3234-4624) lost the ability to infect *Rsv3*-genotype soybean. Such virulence on *Rsv3*-genotype soybean could be restored by the chimeric virus G7(L 3625-4624) where the entire P3 was from G7 (Figs. 2 and 3B). These data suggest P3 is not only a virulence determinant on *Rsv1*- and *Rsv4*-genotype soybeans [11], [39] but is also required for virulence on *Rsv3*-genotype soybean. Thus, P3 is a virulence determinant for all three resistance genes in soybean.

In recent studies, the CI protein of G7H and G5H has been shown to be a virulence and avirulence determinant on *Rsv3*-genotype soybean, respectively [3]. A single amino acid change in the C-terminal of CI was sufficient for G5H to gain virulence on *Rsv3*-genotype soybean. For G2 and G7 pathotypes, both the N and C termini of CI of the N isolate were required for G7 to break down *Rsv3*-resistance [32]. In this report, we not only confirmed that CI is involved in virulence on *Rsv3*-genotype soybean but also provided evidence that CI is essential in virulence on *Rsv1*-soybean (Figs. 2 and 3B). Previously, CI was suggested to be a virulence determinant for several other potyviruses. For instance, the CI protein of TuMV was shown to be responsible for overcoming *TuRB01*- and *TuRB05*-mediated resistance in *Brassica napus* [40], [41]. In the pepper-*Lettuce mosaic virus* (LMV) pathosystem, mutations in the C-terminal region of CI granted an avirulent LMV isolate the ability to infect lettuce carrying either recessive resistance genes *mol1* or *mol2* [41]. Interestingly, a hybrid CI significantly increased the capacity of a *Potato virus Y* (PVY) isolate to break down *pvr2*-mediated resistance in pepper [42].

In addition to the N-terminal P3, we show that the C-terminal moiety of HC-Pro of G7 was required for L, a G2 isolate, to gain virulence on *Rsv1*-genotype soybean and that a point mutation was essential. Our data are consistent with recent findings that concurrent mutations of HC-Pro and P3 are required for G2 (the N isolate) to gain virulence on *Rsv1* -genotype soybean [31], [43]. The HC-Pro of *Potato virus Y* (PVY) has been shown to act as a virulence determinant on potato (*Solanum tuberosum*) and *S. sparsipilum* containing PVY resistance genes *Nc*_tbr_ and *Nc*_spl_, respectively [2]. In several other studies, HC-Pro was found to be involved in potyvirus symptom development [44]–[48].

Multi-viral proteins including HC-Pro, P3 and CI are responsible for potyvirus virulence or avirulence on *Rsv1*-, *Rsv3*- and *Rsv4*-genotype soybeans ([3], [11], [28], [31]–[33], this study) and other *R*-genotype plant species [2], [35]–[38], [40]–[42]. Whether these viral proteins function independently or cooperatively as virulence determinants remains to be elucidated. The function of the potyviral P3 protein is poorly characterized [11]. In addition to its function as a virulence determinant, it has been assumed to play a role in several steps of the potyviral infection cycle such as virus replication, systemic infection, pathogenicity and movement [11], [31], [37], [43], [49]–[52]. Both CI and HC-Pro are multifunctional proteins [15], [43], [53]. CI, having RNA binding, RNA helicase and ATPase activities, has been shown to be essential in virus intra- and intercellular movement and virus replication [54]–[56]. HC-Pro has auto-catalytic proteinase and RNA silencing suppression activities and participates in polyprotein processing, aphid transmission, long-distance movement and viral genome amplification [57]–[59]. Since HC-Pro, P3, 6K1 and CI result from catalytic processing of the large potyviral polyprotein, it is possible that an intermediate precursor protein containing these proteins acts as an elicitor in *Rsv1*-, *Rsv3*- and *Rsv4*-genotype soybeans. As HC-Pro is a cysteine proteinase that efficiently autocleaves the junction between itself and P3, it is more likely that protein complexes formed through protein-protein interactions rather than a single polypeptide play the elicitor role. Indeed, the CI has been shown to bind to other viral proteins including HC-Pro [57], P3 [58] and P3N-PIPO [59]. The essential components of the protein complex may vary depending on the type of resistance. For instance, HC-Pro and P3 are essential for *Rsv1*-resistance [31], [43] whereas P3 and CI are required for *Rsv3*- and *Rsv4*-resistance (this study). As suggested previously [60], overcoming *R*-mediated resistance may be the outcome of a temporal race of the replication and intercellular movement of the invading virus against the host defense response. Therefore, it is also possible that HC-Pro, P3 and CI may operate separately or as a complex with distinct roles for each of them: one as an elicitor to interact with the *R* product and the other(s) as a conditioner to regulate virus replication and intercellular movement. This may explain why the absence of the avirulent elicitor is insufficient and a complementary virulence factor is required for the gain of virulence ([31], [43], this study). The functional roles of these viral proteins are beyond current understanding.

In this study, we show that multiple viral proteins are involved in virulence on the three resistance loci and simultaneous mutations at essential positions of different viral proteins are required for an avirulent SMV to gain virulence on all the resistance loci. In nature, spontaneous mutations in RNA viruses occur during virus replication [61]. It is estimated that the mis-incorporation rates catalyzed by the viral RNA-dependent RNA polymerase (RdRp) are in the range of 10^−4^ to 10^−6^ per nucleotide each generation [9], [62], [63]. For potyviruses such as SMV, frequent identifications of naturally occurring and lab-experimentally evolved resistance-breaking isolates strongly indicate that the mutation rate introduced by the potyviral RdRp can generate a viral population with adequate genetic variability to break down resistance conferred by a single *R* gene in a short time period [8], [11], [25], [31], [43], [48], [64]. These resistance-breaking isolates often require just a single point-mutation [11], [48]. However, as shown in this study, overcoming resistance conferred by two resistance genes, i.e., *Rsv1* and *Rsv4*, requires several concurrent mutations at essential residues of HC-Pro and P3 in a G2 isolate. Very likely, for a G7 isolate to gain virulence on *Rsv3*- and *Rsv4*-genotype soybeans would involve simultaneous mutations on HC-Pro, P3 and CI. The likelihood for an avirulent isolate to have such concurrent mutations is statistically extremely low. Based on this analysis, incorporation of all three resistance genes into a soybean cultivar through gene pyramiding may provide durable resistance to SMV. As such a resistance-pyramided soybean cultivar exerts high selection pressure that may lead the occurrence of a super strain of SMV [65], developing novel genetic resistance to SMV and related viruses remains a long-term challenge for soybean pathologists and breeders.

## Materials and Methods

### SMV isolates, soybean cultivars, inoculation and virus detection

Plasmids containing infectious full-length cDNA clones of SMV isolates L (a G2 isolate), LRB (an L-like naturally evolved *Rsv4*-resistance breaking isolate) and G7 were used as parental viruses [11], [27] to generate G7/G2 chimeric SMVs. Soybean (*Glycine max*) susceptible cultivar Williams 82 (*rsv*) and resistant cultivars PI 96983 (*Rsv1*), L-29 (*Rsv3*) and V94-5152 (*Rsv4*) were grown in a growth chamber with 16-hour light at 22 °C and 8-hour dark at 18 °C. All soybean seeds used in this study were harvested from virus-free plants. Biolistic bombardment of plasmid DNA of parental and chimeric SMVs was initially used to establish SMV infections in Williams 82 [8], [11]. The resulting infected leaf tissues were used as inoculums for pathogenicity tests in soybean cultivars of different genotypes by mechanical inoculation. Virus detection was carried out by double-antibody sandwich enzyme-linked immunosorbent assay (DAS ELISA) and reverse-transcription polymerase chain reaction (RT-PCR) as described previously [8], [11].

### Construction of artificial chimeras between isolates L, LRB and G7 and pathogenicity test

Chimeric SMVs were constructed by using restriction sites indicated (Fig. 1) to swap genomic regions among isolates L, LR and G7 (Fig. 2). For cloning convenience, the full-length cDNA was divided into three fragments designated N, M and C (Fig. 1). Standard DNA manipulation protocols were used for restriction digestions and ligations. DH5α cells (Invitrogen, Burlington, Ontario, Canada) were used for transformation. Plasmid DNA was purified using QIAfilter plasmid midi kit (Qiagen, Toronto, Canada). The purified plasmid was sequenced to confirm identity of the swapped fragment. For pathogenicity tests, each construct was biolistically introduced into three 2-week-old Williams 82 (*rsv*) seedlings. Infected leaves were harvested 15 days post-bombardment and stored in a –80°C freezer for subsequent experiments. The pathogenicity test was repeated three times and each time, four 2-week-old Williams 82 seedlings and 12 *Rsv* soybean plants (four for each of three resistant cultivars described above) were mechanically inoculated as described previously [8], [11].

### Mutagenesis of HC-Pro

The BglII-KpnI fragment of the L infectious cDNA plasmid was PCR amplified using two pairs of primers containing mutations wherever necessary to generate two PCR products. The amplicons were gel-purified with a QIAquick gel extraction kit (Qiagen, Toronto, Canada) and the purified PCR-products were used as templates to produce a single PCR product with a pair of primers containing BglII and KpnI restriction sites. The PCR products containing a single mutation (C720Y) or double mutations (R682M, C720Y) were digested with BglII and KpnI and cloned into L(G7 2342-3237)(G1054R) (Fig. 2). The resulting plasmids L(G7 2342-3237)(C720Y G1054R) and L(G7 2342-3237) (R682M, C720Y G1054R) were sequenced to confirm mutation(s) and then used for pathogenicity tests.

### DAS ELISA, RNA isolation, RT-PCR and sequencing

Virus detection was carried out by DAS ELISA and RT-PCR as described [8], [11]. Approximately 100 mg of leaf from each soybean seedling was sampled at 14, 28 and 42 dpi into an eppendorf tube and flash frozen in liquid nitrogen. The extraction buffer and other ELISA buffers were prepared as described for an SMV DAS ELISA kit (Agdia, Elkhart, Indiana, USA) using alkaline phosphatase conjugated antibodies. For SMV detection by RT-PCR, total RNA was extracted [8] and RT-PCR was performed using two sets of primers (one for SMV and the other for EF 1a serving as a control). SMV mutants were verified by sequencing either PCR products or cloned cDNA as described [8], [11].

### Isolation and sequencing of *Rsv4*-resistance breaking isolate

All recombinant SMVs were maintained in Williams 82 as an inoculum source. After three passages, an *Rsv4*-genotype soybean seedling inoculated with L (G7 2342-3237) showed typical SMV symptoms and was positive in ELISA and RT-PCR assays. The virus was purified, cloned and completely sequenced essentially as described [8], [11]. The complete genome sequence of this virus was deposited into GenBank with accession number JN416770.

## Supporting Information

Figure S1 **Amino acid sequence alignment of the SMV 6K1 protein.**
(TIF)

Figure S2 **Amino acid sequence alignment of the SMV P3N-PIPO protein.** Translational frameshift/slippage is indicated by an arrow.
(TIF)

Figure S3 **Amino acid sequence alignment of the N-terminal moiety of the SMV CI protein.** Restriction site AgeI is indicated.
(TIF)

Figure S4 **Amino acid sequence alignment of the C-terminal moiety of the SMV CI protein.**
(TIF)
