# Supplementary figures and images for: Simultaneous Mutations in Multi-Viral Proteins Are Required for Soybean mosaic virus to Gain Virulence on Soybean Genotypes Carrying Different R Genes
Source: PLoS One. 2011 Nov 30;6(11):e28342. doi: 10.1371/journal.pone.0028342 (PMC3227670; doi:10.1371/journal.pone.0028342)

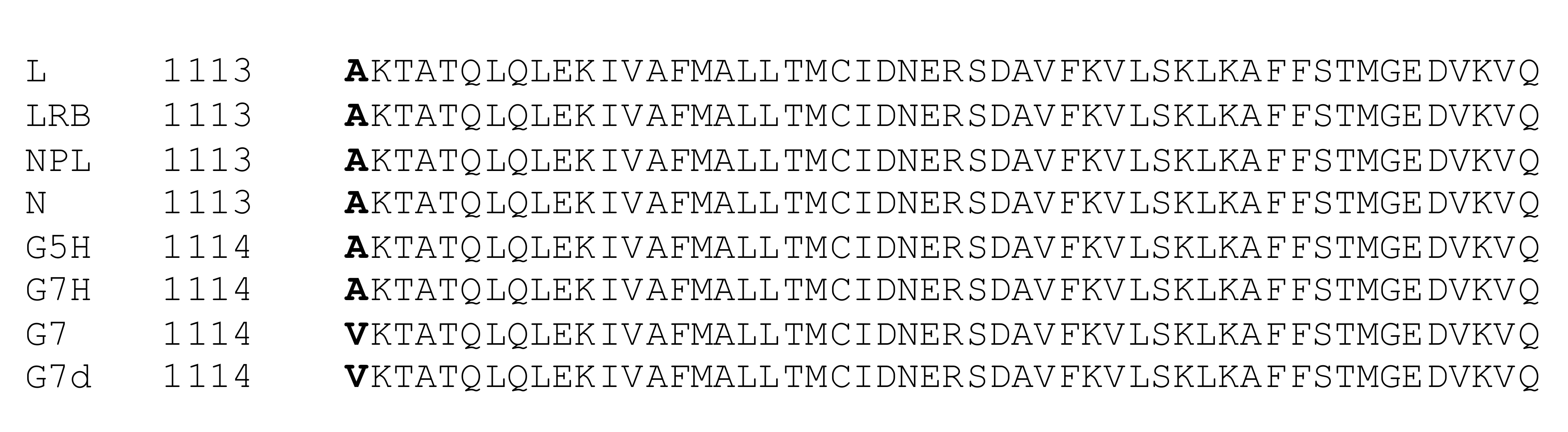

Supplement: Figure S1 — Amino acid sequence alignment of the SMV 6K1 protein. (TIF) [file pone.0028342.s001.tif]

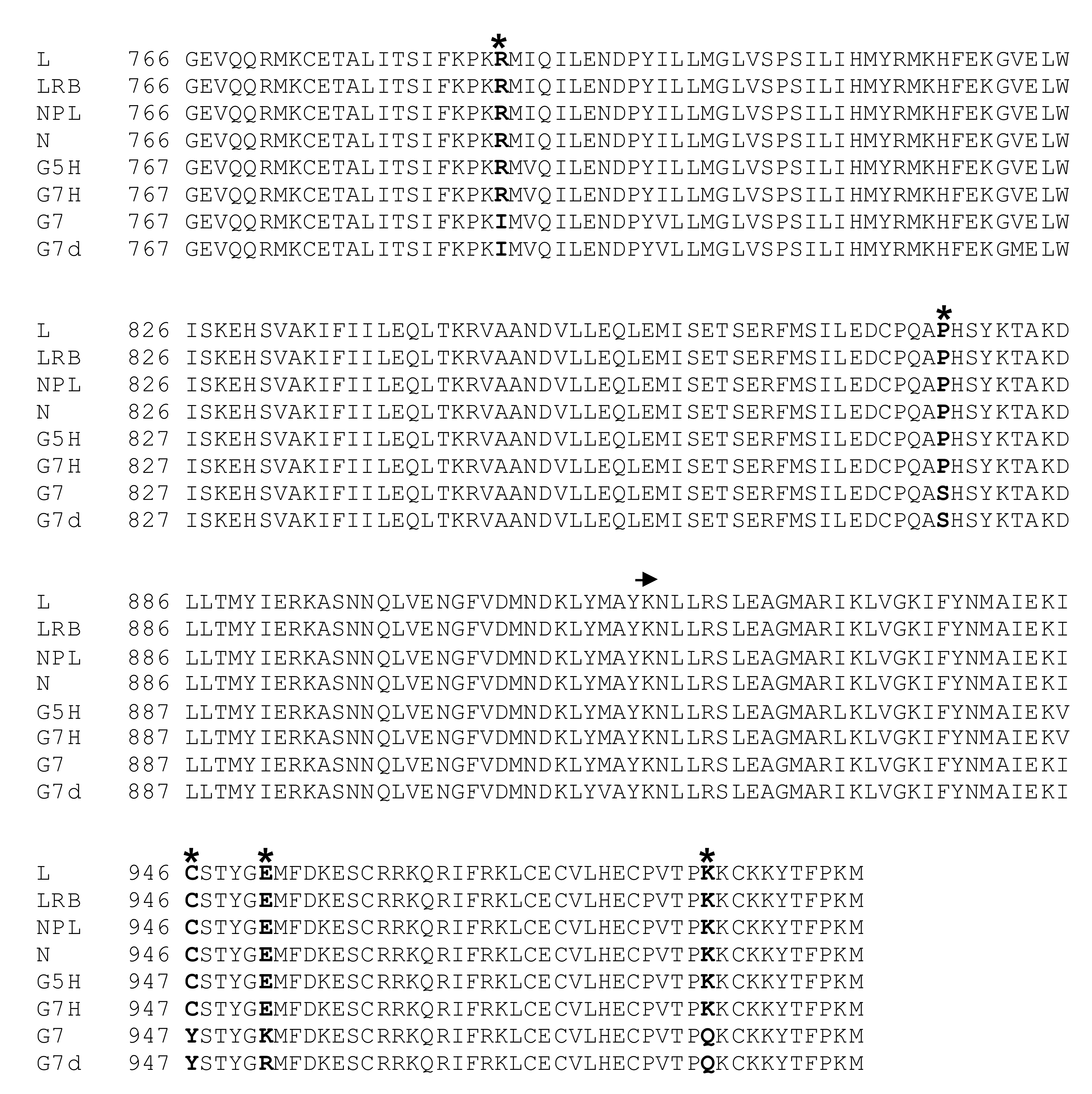

Supplement: Figure S2 — Amino acid sequence alignment of the SMV P3N-PIPO protein. Translational frameshift/slippage is indicated by an arrow. (TIF) [file pone.0028342.s002.tif]

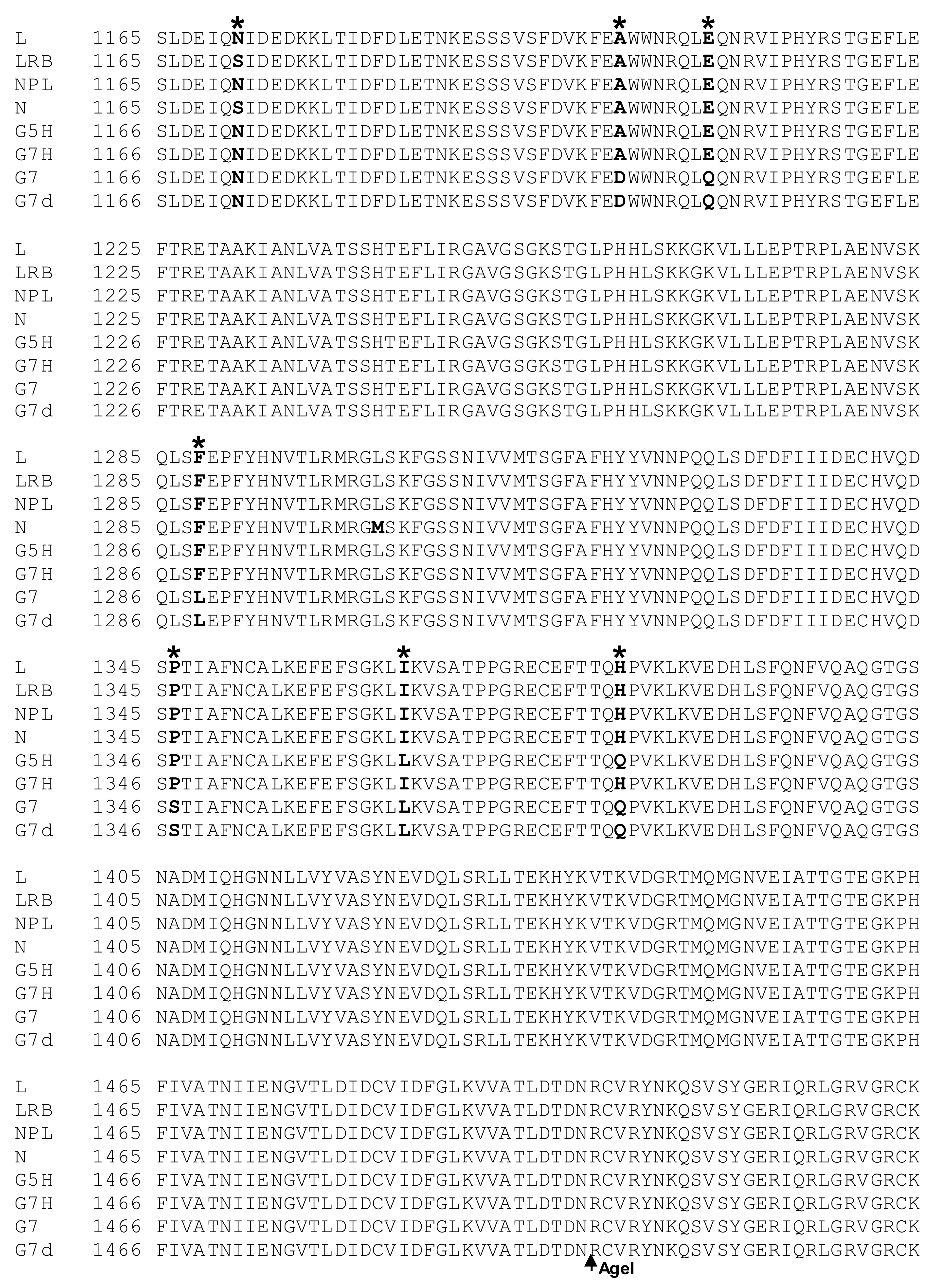

Supplement: Figure S3 — Amino acid sequence alignment of the N-terminal moiety of the SMV CI protein. Restriction site AgeI is indicated. (TIF) [file pone.0028342.s003.tif]

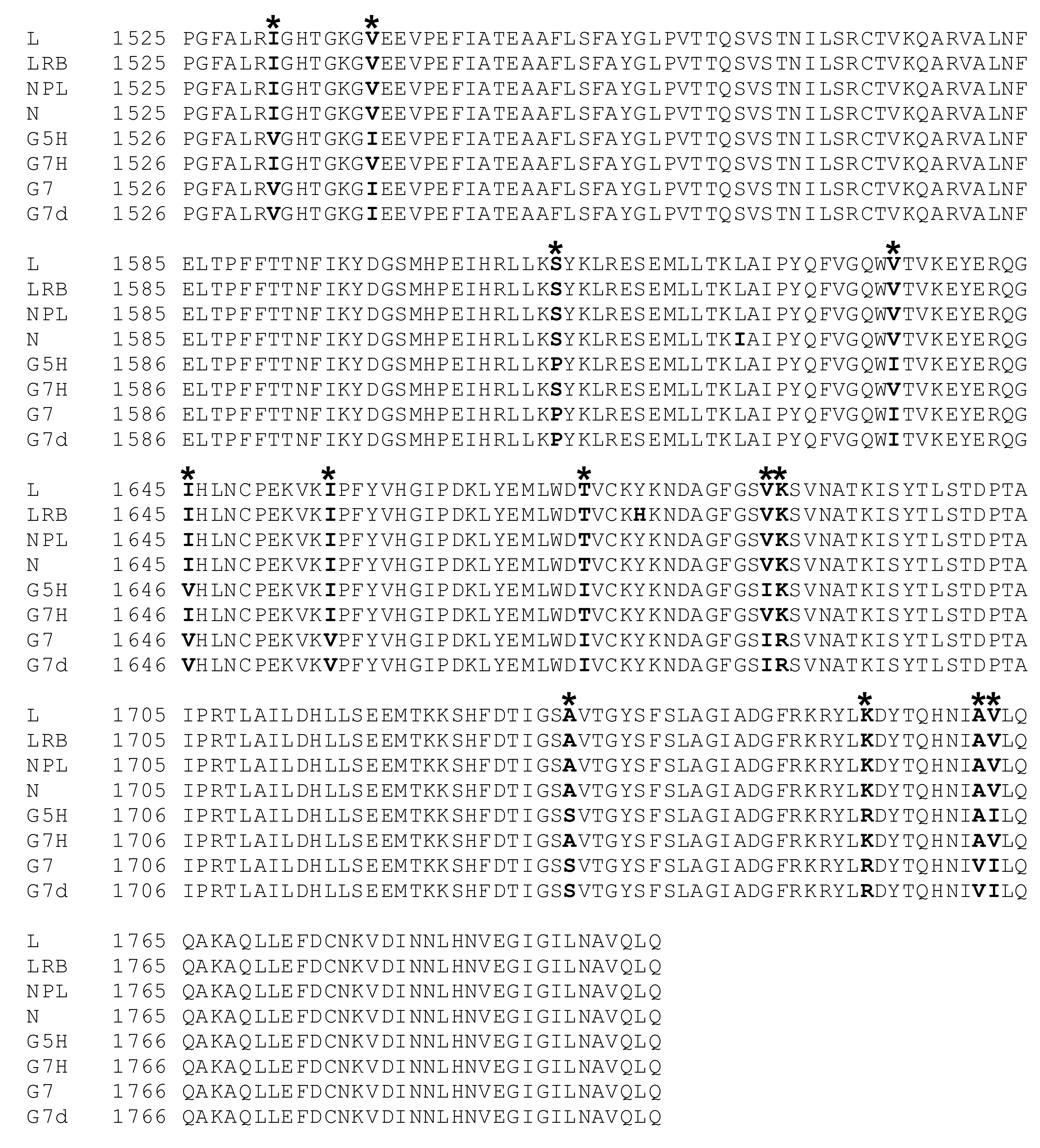

Supplement: Figure S4 — Amino acid sequence alignment of the C-terminal moiety of the SMV CI protein. (TIF) [file pone.0028342.s004.tif]
